# Supplementary material for: Transcranial Photosensitizer-Free Laser Treatment of Glioblastoma in Rat Brain
Source: Int J Mol Sci. 2023 Sep 5;24(18):13696. doi: 10.3390/ijms241813696 (PMC10530910; doi:10.3390/ijms241813696)
Supplement: Supplementary file 1 [file ijms-24-13696-s001.zip › ijms-2557856-supplementary.pdf]

Supplementary Materials

Transcranial photosensitiser-free laser treatment of glioblastoma in rat brain

Oxana Semyachkina-Glushkovskaya [1,2*], Sergey Sokolovski [3*], Ivan Fedosov[4], Alexander Shirokov[2,5], Nikita Navolokin[2,6], Alla Bucharskaya[6], Inna Blokhina[2], Andrey Terskov[2], Alexander Dubrovski[4], Valeria Telnova[2], Anna Tzven[2], Maria Tzoy[4], Arina Evsukova[2], Daria Zhlatogosrkaya[2], Viktoria Adushkina[2], Alexander Dmitrenko[2], Maria Manzhaeva[2], Valeria Krupnova[2], Alessio Noghero [7], Denis Bragin[7,8], Olga Bragina[7,8], Ekaterina Borisova[9], Jürgen Kurths[1,2,10,11], Edik Rafailov[3]

[1] Physics Department, Humboldt University, Newtonstrasse 15, 12489 Berlin, Germany; glushkovskaya@mail.ru; juergen.kurths@pik-potsdam.de

[2] Department of Biology, Saratov State University, Astrakhanskaya Str. 83, 410012 Saratov, Russia; glushkovskaya@mail.ru; shirokov_a@ibppm.ru; nik-navolokin@yandex.ru; inna-474@yandex.ru; terskow.andrey@gmail.com; ler.vinnick2012@yandex.ru; anna.kuzmina.270599@mail.ru; arina-evsyukova@mail.ru; eloveda@mail.ru; adushkina.info@mail.ru; admitrenko2001@mail.ru; mariamang1412@gmail.com; krupnova_0110@mail.ru; juergen.kurths@pik-potsdam.de

[3] Optoelectronics and Biomedical Photonics Group, AIPT, Aston University, Birmingham, UK; s.sokolovsky@aston.ac.uk; e.rafailov@aston.ac.uk

[4] Physics Department, Saratov State University, Astrakhanskaya Str. 83, 410012 Saratov, Russia; fedosov_optics@mail.ru; paskalkamal@mail.ru; dethaos@bk.ru;

[5] Institute of Biochemistry and Physiology of Plants and Microorganisms, Russian Academy of Sciences, Prospekt Entuziastov 13, Saratov 410049, Russian Federation; shirokov_a@ibppm.ru

[6] Department of Pathological Anatomy, Saratov Medical State University, Bolshaya Kazachaya Str. 112, Saratov 410012, Russia; nik-navolokin@yandex.ru; allaalla_72@mail.ru

[7] Lovelace Biomedical Research Institute, Albuquerque, NM 87108, USA; noghero@gmx.com; dbragin@salud.unm.edu; obragina@gmx.com

[8] Department of Neurology, School of Medicine, University of New Mexico, , Albuquerque, NM 87131, USA; dbragin@salud.unm.edu; obragina@gmx.com

[9] Institute of Electronics, Bulgarian Academy of Sciences, Tsarigradsko Chaussee Blvd. 72, Sofia 1784, Bulgaria; ekaterina.borisova@gmail.com

[10] Potsdam Institute for Climate Impact Research, Telegrafenberg A31, 14473 Potsdam, Germany; juergen.kurths@pik-potsdam.de

[11] Systems, Sechenov First Moscow State Medical University Mowcow, Russia; juergen.kurths@pik-potsdam.de

* Correspondence: O S-G glushkovskaya@mail.ru; Tel.: +78452519220; S S s.sokolovsky@aston.ac.uk (+440121 204 3718)

Citation: To be added by editorial staff during production.

Academic Editor: Firstname Lastname

Received: date
Revised: date
Accepted: date
Published: date

Copyright: © 2023 by the authors. Submitted for possible open access publication under the terms and conditions of the Creative Commons Attribution (CC BY) license (https://creativecommons.org/licenses/by/4.0/).

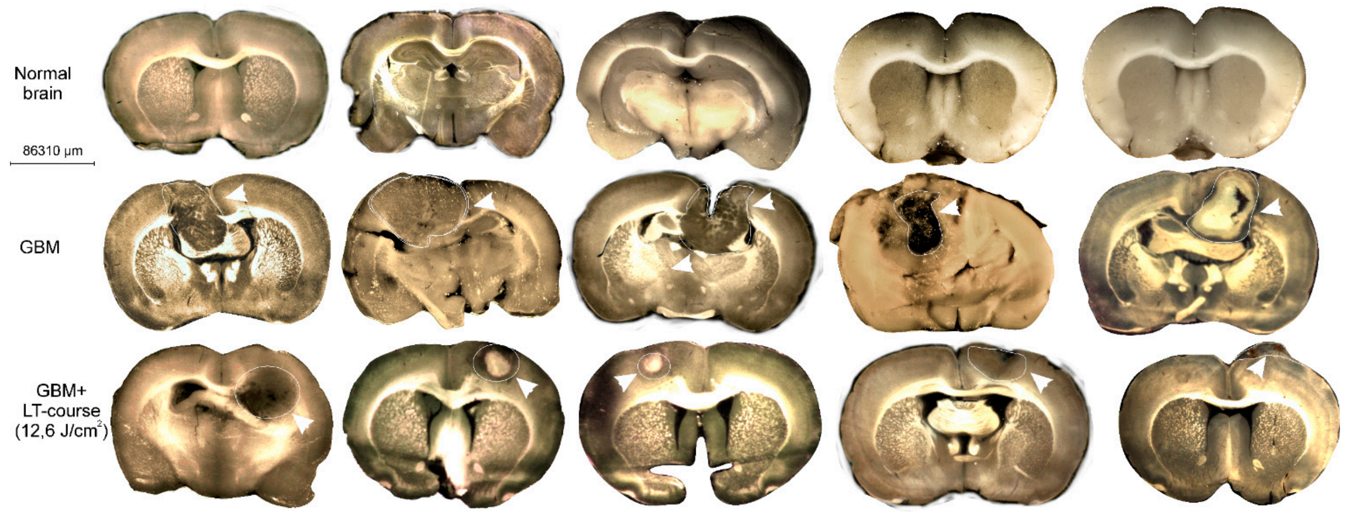

Figure S1. Representative 2D images of the normal rat brain (at the top), 4 weeks of GBM growth without (middle) and after the 12.6 J/cm^2 LT (on the bottom).

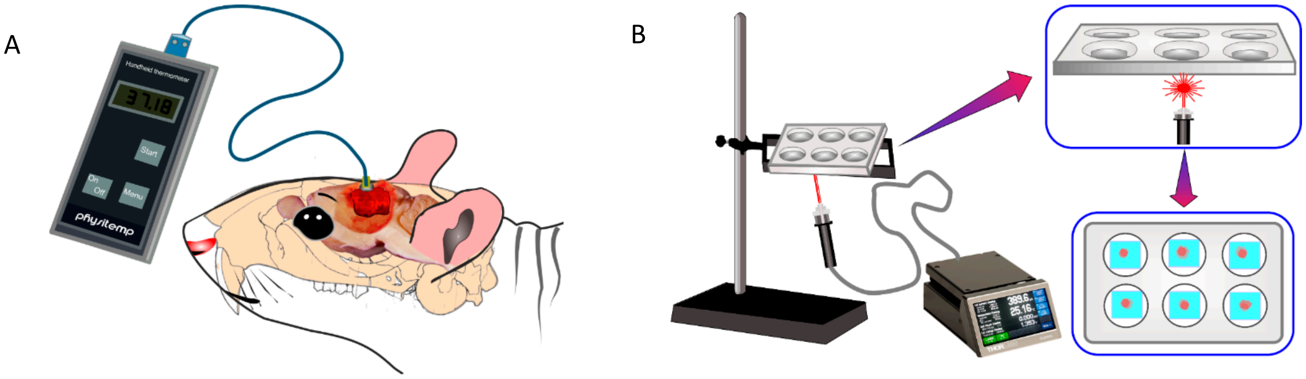

Figure S2. Schematic illustration of *in vivo* temperature measurements on the surface of the rat brain with mature GBM (**A**). Schematic illustration of NIR laser irradiation applied to the C6 glioma cell cultures in 6-wellplates (**B**). Since mature GBM reaches the cortex surface and appears under the skull on the surrounding intact brain tissues. The optical setup was developed based on the use of 1267 nm laser (LD-1267-FBG-350, Innolume, Dortmund, Germany, laser driver: THORLABS CLD1015) with collimator (Thorlabs Ltd., USA) given 5 mm diameter beam on the illuminated surface of the rat skull. The scattering coefficient and diffuse transmittance were measured on the freshly harvested skull bone of 8 weeks old rats. The confluent (70-80%) C6 glioma cell cultures on coverslips were irradiated with the same laser setup as described above.

Table S1. - Temperature (^{0}C) at the external surface of skull and the brain cortex before and after LT in rats with GBM of 4 weeks growth

№ group	Number of rat/ Thermo-couple posi-tioning	1	2	3	4	5	6	7	8	9	10	Mean±SEM/	Mean±STD	Mean±SEM	Welch test
no LT															
1		35.49	35.57	35.68	35.74	36.46	35.31	36.26	36.15	35.84	35.28	35.77±0.13	35,78±0,40	35,78±0,13	
2	Under the skull on the cortex surface	37.25	37.31	37.18	37.28	37.29	37.11	36.27	37.23	37.32	37.26	37.15±0.31	37,15±0,32	37,15±0,10	
70 mW															
3	The skull external surface	36.28	36.15	36.21	36.21	36.05	35.63	36.07	36.52	35.71	35.39	36.02±0.34	36,022±0,34	36,022±0,11	Comparison with №1 NO differences P=0,1607
4	Under the skull on the cortex surface	37.00	37.71	37.12	37.49	36.71	36.55	36.81	37.22	38.45	36.22	37.13±0.82	37,13±0,64	37,123±0,20	Comparison with №2 NO differences P=0,9237
100 mW															
5	The skull external surface	36.48	36.44	36.38	36.52	36.09	36.55	36.62	36.67	39.45	39.00	37.02±1.14	37,02±1,18	37,02±0,37	Comparison with №1 Different P<0.01 P= ,00907
6	Under the skull on the cortex surface	37.34	37.01	37.24	37.11	37.03	36.98	36.84	36.92	37.39	37.99	37.18±0.32	37,19±0,33	37,19±0,11	Comparison with №2 NO differences P=0,8126
130 mW															
7	The skull external surface	37.69	37.84	37.19	37.68	37.48	38.17	37.88	38.10	38.53	38.99	37.95±0.52	37,96±0,52	37,96±0,16	Comparison with №1 Different P=8,39984E-9

8	Under the skull on the cortex surface	39.13	39.21	38.56	39.22	39.03	39.45	38.84	39.42	39.15	38.15	39.01±0.4	39,02±0,40	39,02±0,13	Comparison with №2 Different p<0.01

Table S2. – The statistical analysis of survival rate in the GBM and GBM+LT (12.6 kJ/cm^2) groups and in the LV ligation group

**The name of test**	**X2 test**	**Significance**
between the GBM and GBM+LT (8.7 kJ/cm^2)		
Log Rank (Mantel-Cox)	2.2E-2	0.88
Breslow (Generalized Wilcoxon)	6E-3	0.94
Tarone-Ware	1E-3	0.97
between the GBM and GBM+LT (12.6 kJ/cm2)		
Log Rank (Mantel-Cox)	18.82	1.4E-5
Breslow (Generalized Wilcoxon)	35.7	2.3E-09
Tarone-Ware	29.09	6.9E-08
between the GBM and GBM+LT (16.3 kJ/cm2)		
Log Rank (Mantel-Cox)	14.35	1.6E-4
Breslow (Generalized Wilcoxon)	8.45	3.6E-3
Tarone-Ware	10.93	9.5E-4
Between the GBM group and the LV ligation group		
Log Rank (Mantel-Cox)	48.35	3.6E-12
Breslow (Generalized Wilcoxon)	41.03	1.5E-10
Tarone-Ware	44.90	2.1E-11

n=30 in each group

Table S3. – The effects of LT-course with different laser doses on the 2-weeks and 4-weeks old GBM volume (mm^3)

**Number of rats**	**1**	**2**	**3**	**4**	**5**	**6**	**7**	**8**	**9**	**10**	**Mean± SEM**	**Welch's test**
no LT 2 weeks old GBM	78	112	90	105	87	80	82	117	79	82	91±4,6	
no LT 4 weeks old GBM	201	243	212	209	197	261	263	199	202	264	225±9,7	
8.7 J/cm² 2 weeks old GBM	69	81	132	100	93	89	96	98	88	95	94±5,1	T=-0,42 P=0,68
8.7 J/cm² 4 weeks old GBM	288	315	310	309	274	245	118	135	173	166	233±25,5	T=0,31 P=0,75
12.6 J/cm² 2 weeks old GBM	128	80	107	51	98	90	83	64	98	92	89±6,8	T=-0,254 P=0,8
12.6 J/cm² 4 weeks old GBM	203	200	196	112	172	158	154	115	101	112	152±12,7	T=4,65 P<0.001
16.3 J/cm² 2 weeks old GBM	95	82	67	98	71	82	99	107	68	81	85±4,46	T=0.96 P=0.34
16.3 J/cm² 4 weeks old GBM	108	185	207	111	183	197	184	222	112	81	159±15,9	T=3.6 P=0.002

The comparison between: no LT 2 weeks old GBM and 8.7; 12.6; 16.3 J/cm²/ 2 weeks old GBM; no LT for 4 weeks old GBM and 8.7; 12.6; 16.3 J/cm²/ 4 weeks old GBM

Table S4. – The effects of 12.6 J/cm^2 LT on the 1-week and 4-weeks old GBM volume (mm^3)

**Number of rats**	**1**	**2**	**3**	**4**	**5**	**Mean±SEM/ Test**	**Welch's test**
no LT 1 weeks old GBM	6	12	27	6	1	10±4	
no LT 4 weeks old GBM	149	349	174	232	225	225±77	
12.6 J/cm^{2}1 week old GBM	47	6	3	1	6	12±8	T=-0,226 P=0,827
12.6 J/cm^2 4 weeks old GBM	102	108	50	76	163	99±48	T=3,2 P=0,013

The comparison between: no LT 1 week old GBM and 12.6 J/cm^2/ 1 week old GBM; no LT 4 week old GBM and 12.6 J/cm^2/ 4 week old GBM

Table S5. – The changes of the expression of markers of apoptosis, autophagy, proliferation and vacuoles formation in sham rats and in rats with GBM without and after LT-course (12.6 kJ/cm^2 laser irradiation)

**The tested markers**	**Sham group, no course**	**Sham group + LT course**	**GBM group, no LT-course**	**GBM group + LT course**	**Welch's test. Comparison between GBM no LT course and GBM + LT course, mean±SEM, n=20 in each group**
Cell number in the view field (x774)	29.0±1.9	28.1±1.9	67.0±3.2	112.2±7.7	
Nucleus diameter, μm	8.5±0.4	8.7±0.4	9.2±0.3	7.6±4.7	
Ki67	0.0±0.0	0.0±0.0	37.5±1.97	32.2±2.4	T=22,3, p<0.01
Bax	0.0±0.0	0.0±0.0	7.1±1.1	95.8±2.1	T=174,3,
p53	0.0±0.0	0.0±0.0	0.0±0.0	31.5±4.9	T=43,1, p<0.01
Lc3b	100.0±0.0	100.0±0.0	95.1±1.8	26.4±5.14	T=74,8, p<0.01
Fas	0.0±0.0	0.0±0.0	0.0±0.0	1.92±1.32	T=7,7, p<0.01
Clathrin	0.0±0.0	0.0±0.0	94.2±2.0	23.6±6.4	T=117,8, p<0.01
Caveolin	0.0±0.0	0.0±0.0	18.4±1.8	7.0±2.4	T=17,6, p<0.01

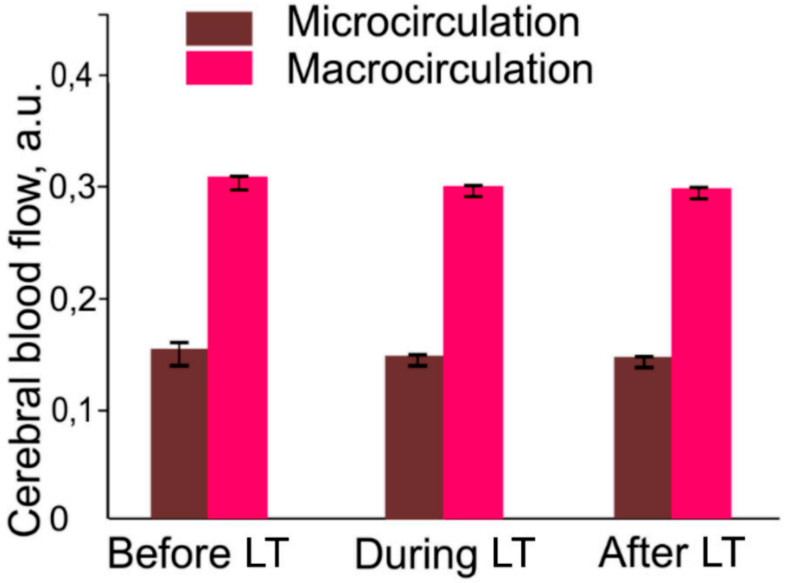

Figure S3. The changes in the cerebral blood flow before and after 12.6 kJ/cm² LT course (mean±SEM), n=10 in each group.

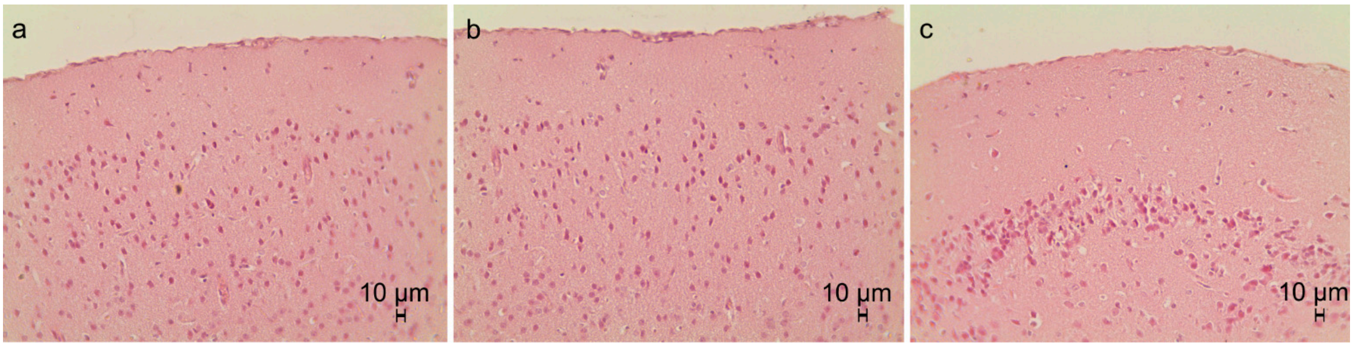

Figure S4. The histological analysis of the brain tissues before (a), during (b) and after 12.6 kJ/cm²LT course, n=10 in each group

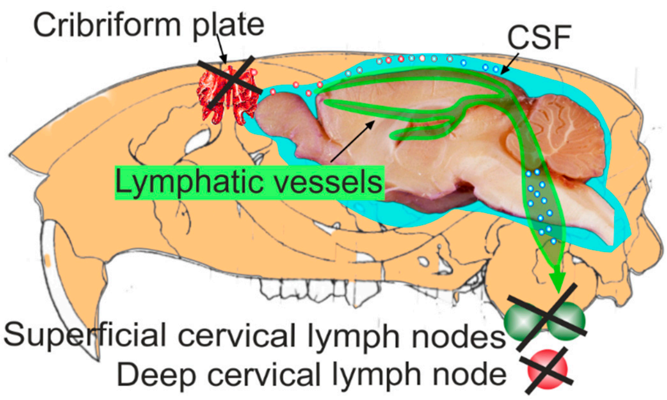

Figure S5. Scheme of blocking of the cerebrospinal fluid (CSF) outflow through the cribriform plate and the cervical lymphatic vessels that is accompanied by the accumulation of extensive CSF in the brain.

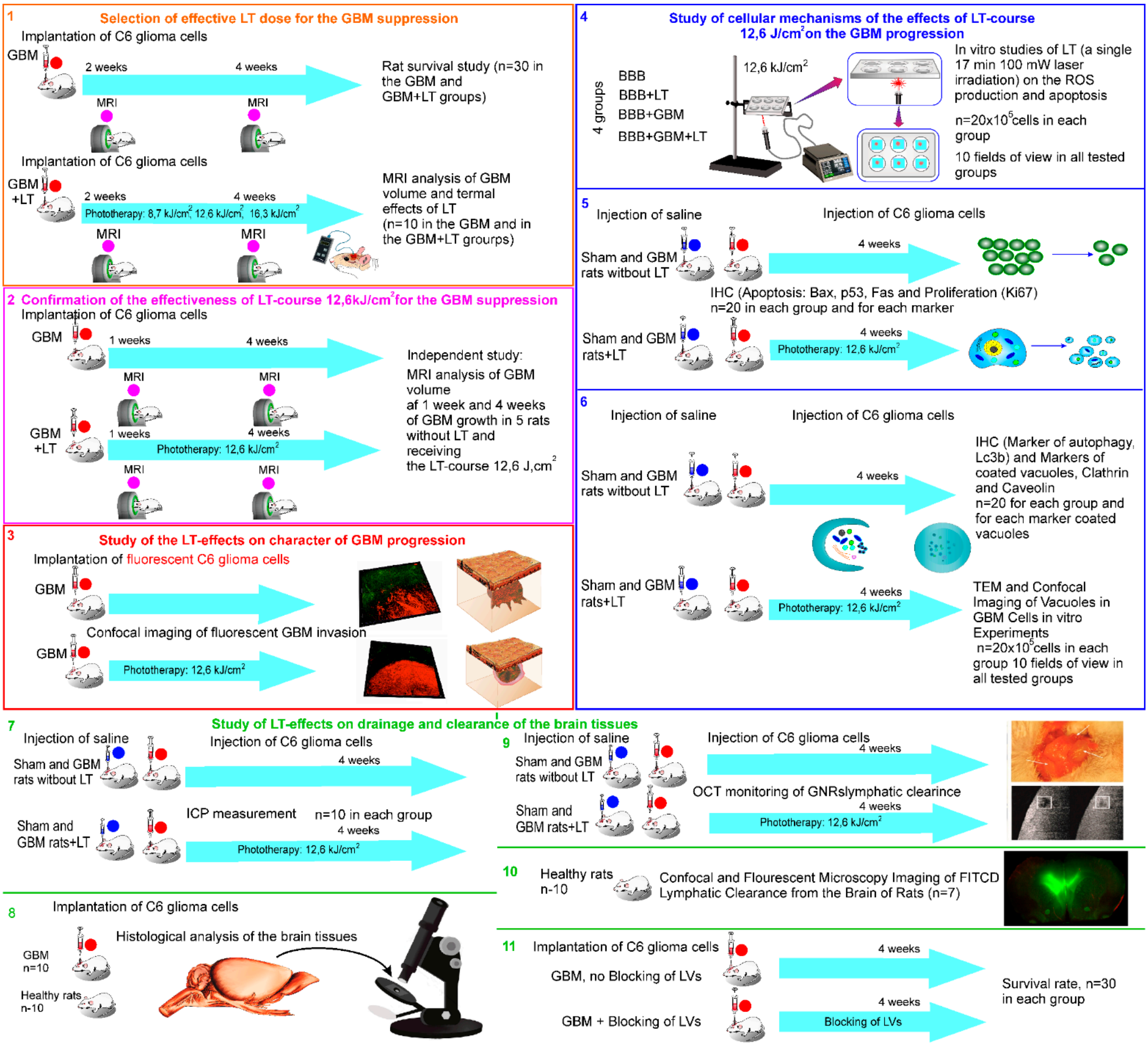

Figure S6. Design of experiments to study of LT effects on GMB growth and mechanisms of LT-induced suppression of GBM progression.

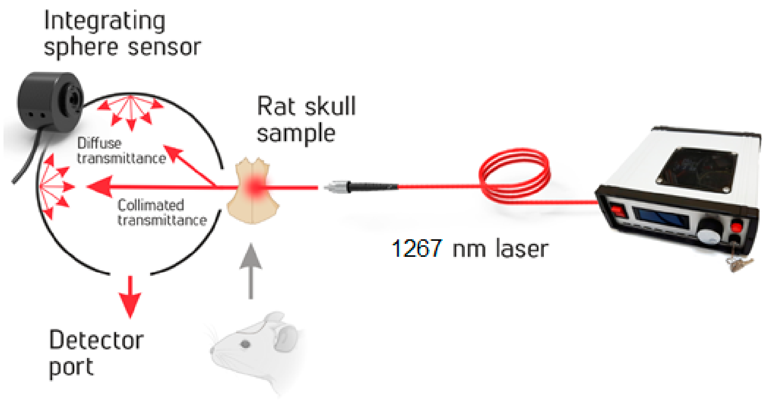

Figure S7. Virtual scheme of the optic set up for measuring rat skull transmittance level for 1267 nm QD semiconductor laser.
